# Tumor-Derived Extracellular Vesicles Activate Normal Human Fibroblasts to a Cancer-Associated Fibroblast-Like Phenotype, Sustaining a Pro-Tumorigenic Microenvironment

**DOI:** 10.3389/fonc.2022.839880

**Published:** 2022-02-23

**Authors:** Ilaria Giusti, Marianna Di Francesco, Giuseppina Poppa, Letizia Esposito, Sandra D’Ascenzo, Vincenza Dolo

**Affiliations:** Department of Life, Health and Environmental Sciences, University of L’Aquila, L’Aquila, Italy

**Keywords:** extracellular vesicles, cancer-associated fibroblasts, CAFs, ovarian cancer, tumor microenvironment, vesicles subpopulations

## Abstract

Fibroblasts in the tumor microenvironment have been proven to actively participate in tumor progression; they can be “educated” by cancer cells acquiring an activated state and, as such, are identified as cancer-associated fibroblasts (CAFs); CAFs, in turn, remodel tumor stroma to be more advantageous for cancer progression by modulating several processes, including angiogenesis, immunosuppression, and drug access, presumably driving the chemoresistance. That is why they are believed to hamper the response to clinical therapeutic options. The communication between cancer cells and fibroblasts can be mediated by extracellular vesicles (EVs), composed of both exosomes (EXOs) and microvesicles (MVs). To verify the role of different subpopulations of EVs in this cross-talk, a nearly pure subpopulation of EXO-like EVs and the second one of mixed EXO- and MV-like EVs were isolated from ovarian cancer cells and administered to fibroblasts. It turned out that EVs can activate fibroblasts to a CAF-like state, supporting their proliferation, motility, invasiveness, and enzyme expression; EXO-like EV subpopulation seems to be more efficient in some of those processes, suggesting different roles for different EV subpopulations. Moreover, the secretome of these “activated” fibroblasts, composed of both soluble and EV-associated molecules, was, in turn, able to modulate the response of bystander cells (fibroblasts, tumor, and endothelial cells), supporting the idea that EVs sustain the mutual cross-talk between tumor cells and CAFs.

## Introduction

The term “extracellular vesicles” (EVs) is used to describe all spherical and membrane-enclosed vesicles released into the extracellular space by both normal and tumor cells (1). When their size and cellular origin are considered, it is possible to distinguish three subpopulations of EVs: exosomes (EXOs), microvesicles (MVs), and apoptotic bodies (ABs) (2).

ABs are released from the plasma membrane as blebs when cells undergo apoptosis and have a size ranging between 1 and 4 µm in diameter. EXOs and MVs are released, instead, from viable cells; EXOs are the smallest EVs, ranging from 40 to 150 nm in diameter, and originate from the formation of an early endosome at the plasma membrane and the subsequent maturation into multivesicular bodies, where intraluminal vesicles (ILVs) form in the lumen by inward budding of the membrane; their final fusion with the plasma membrane results in the release of the ILVs into the extracellular space originating EXOs. MVs are larger than EXOs, being around 100 nm to 1 µm in size, and originate directly from the outward budding of the plasma membrane (2).

To date, EVs are considered as an intercellular communication mechanism acting as molecular shuttles packaged with a bioactive cargo of proteins, lipids, and nucleic acids that are used by cells to interact with the neighboring ones to modulate their environment (3, 4); once released, indeed, they can interact with target cells, releasing their content into extracellular space following EV lysis, interacting with their receptors, by fusion, or other mechanisms yet to be identified (3–5).

As such, EVs are involved in many physiological and pathological processes (6–9); among the latter, cancer has been the focus in the past years given the cancer-derived EV involvement in many tumor-related processes such as angiogenesis induction, invasion, motility, evasion from immune surveillance, apoptosis escape, and drug resistance promotion (10–17).

Over the last few years, some evidence has emerged suggesting that, during cancer progression, EVs are also able to support the creation of a microenvironment encouraging cancer growth, progression, and metastasis by conveying messages to nearby stromal cells, including the so-called “cancer-associated fibroblasts” (CAFs) (18, 19).

CAFs, along with the extracellular matrix and several cell types (including endothelial cells, immune cells, and adipocytes), constitute the tumor stroma in many types of cancer, including ovarian cancer. In this kind of tumor, the stroma could account for a large percentage of tumor tissue (up to 83%), leading to hypothesize a relevant role for CAFs (20, 21). CAFs have been demonstrated to actively participate in cancer progression, being involved in cancer metastasis, angiogenesis stimulation, immunosuppression induction, and drug resistance (22–24).

Our previous study has demonstrated that the human ovarian cancer cell line CABA I releases different EV subpopulations in a time-dependent mode; starved CABA I cells, indeed, once stimulated with fetal bovine serum (FBS), released a first nearly pure population of EXO-like EVs (mean size ~100 nm) and a second one mix of EXO- and MV-like EVs (size > 100 nm) (25). These data highlighted that different time intervals lead to the release of different subpopulations of EVs, in terms of not only size but also amount and molecular composition, suggesting possible different cargoes and, consequently, different biological roles for the different subpopulations.

This work aimed to verify if specific EV subpopulations released from CABA I were able to activate normal human fibroblasts into CAF-like cells and to verify the effect of such activation on surrounding cells (cancer cells, endothelial cells, and not activated fibroblasts).

Our present findings support the idea that ovarian cancer cells can modulate fibroblast behavior through the release of EVs, activating them to a CAF-like state that is able, in turn, to stimulate the nearby cells. However, the different subpopulations of EVs show a different ability to stimulate these processes: the EXO-like EVs rather than the mixed population of EXO- and MV-like EVs seem to be more efficient in some activation processes. Overall, these findings suggest that EVs, particularly EXOs, can be considered pivotal targets of novel anticancer therapies to hamper fibroblast activation.

## Materials and Methods

### Cell Cultures

CABA I cell line was established from the ascitic fluid of an ovarian carcinoma patient not undergoing drug treatment (26). Cells were grown as monolayers in Roswell Park Memorial Institute-1640 (RPMI-1640) supplemented with 5% (v/v) heat-inactivated FBS, 1× penicillin/streptomycin, and 2 mM of l-glutamine.

Normal human dermal fibroblasts (NHDF) cell line was purchased from Lonza (Walkersville, MD, USA) and grown as a monolayer in Dulbecco’s modified Eagle medium (DMEM) supplemented with 10% (v/v) heat-inactivated FBS, 2 mM of l-glutamine, penicillin, and streptomycin. Cells were subcultured and used within the 15th doubling, as suggested by Lonza’s protocols.

Human umbilical vein endothelial cells (HUVECs) were isolated from human umbilical cord veins; the study was conducted in accordance with the Declaration of Helsinki, approved by the Internal Review Board of L’Aquila University (protocol code 07/2018, February 2018), and informed consent was obtained from all subjects involved. Endothelial cells were grown on 1% gelatin-coated flasks in DMEM supplemented with 10% (v/v) heat-inactivated FBS, 10% (v/v) heat-inactivated newborn calf serum (NCS), 20 mM of HEPES [*N*- (2-hydroxyethyl) piperazine-*N*′- (2-ethane sulfonic acid)], 6 U/ml of heparin, 2 mM of l-glutamine, 50 µg/ml of endothelial cell growth factor (ECGF), penicillin, and streptomycin. These cells were used within the fifth passage.

All cell lines were cultured at 37°C in a humidified atmosphere with 5% CO_2_, and experiments were carried out on sub-confluent (except for wound-healing assays) and mycoplasma-negative cells.

FBS, RPMI, DMEM, glutamine, penicillin, and streptomycin were purchased from Euroclone (Euroclone SpA, Milan, Italy); Hepes and ECGF were from Sigma-Aldrich (St. Louis, MO, USA); and NCS was from Gibco (Gibco, Thermo Fisher Scientific, Waltham, MA, USA).

### Extracellular Vesicle Isolation From Culture Media

The protocol to isolate the two different EV subpopulations, used to stimulate NHDF, had been previously set (25). Briefly, CABA I cells were starved in serum-free medium for 24 h to avoid EV release and subsequently stimulated with 5% of 40-nm-filtered FBS HyClone (Thermo Scientific, Rockford, IL, USA) in RPMI-1640; conditioned media (CMs) containing EVs were collected in sterile working conditions after 30 min and 18 h from the HyClone supplement.

To isolate EVs, these CMs were firstly centrifuged at 4°C at 600×*g* for 15 min and then at 1,500×*g* for 30 min to remove cells and large debris, respectively. The resulting supernatants were centrifuged at 100,000×*g* (Rotor 70Ti, Quick-Seal Ultra-Clear tubes, k_adj_ 221, brake 9) for 2 h at 4°C in an Optima XPN-110 Ultracentrifuge (Beckman Coulter, Brea, CA, USA). For each preparation, the EVs were derived from a starting cell number of 4,500,000–5,000,000 cells for 30-min collection and 7,500,000–9,000,000 for the 18-h collection. Isolated vesicles were resuspended in Dulbecco’s phosphate-buffered saline (PBS) (EuroClone, Milan, Italy), and the determination of vesicle quantification was carried out by measuring the vesicle-associated protein levels using the Bradford method (27) (Bio-Rad, Milan, Italy) with bovine serum albumin (BSA; Sigma-Aldrich, St. Louis, MO, USA) as the standard.

The EV subpopulations obtained with this experimental protocol have already been previously characterized by markers, NanoSight assay, and transmission electron microscopy (25). Hereinafter, EVs from CMs collected after 30 min and 18 h from the HyClone supplement will be indicated, respectively, as EVs_30′_ and EVs_18h_.

### Fibroblast Treatments With EVs_30′_ and EVs_18h_

NHDF were administered with EVs_30′_ and EVs_18h_ by supplying 1 µg of EVs/ml every day for up to 5 days, in a cumulative way: EVs were added every 24 h without replacing the medium for the entire duration of the treatment, so as to mimic the continuous release of EVs by cancer cells within the tumor microenvironment and the persistent exposure of fibroblasts to EVs. Treatments were performed by adding the EVs to culture media supplemented with a reduced percentage of FBS (2%) to limit the serum stimulatory effect while ensuring fibroblast survival.

Hereinafter, NHDF treated with EVs_30′_ and EVs_18h_ will be, respectively, indicated as NHDF_30′_ and NHDF_18h_. Untreated fibroblasts will be indicated as NHDF.

### Western Blotting

To verify the NHDF activation into a CAF-like state, 48 h after the end of a 5-day treatment with EVs, NHDF, NHDF_30′_, and NHDF_18h_ were washed three times with PBS and lysed in radioimmunoprecipitation assay (RIPA) Lysis Buffer, containing 50 mM of Tris-HCl, pH 7.5, 150 mM of NaCl, 0.5% sodium deoxycholate, 1% Triton-X, 0.1% sodium dodecyl sulfate (SDS), 5 mM of EDTA, 100 mM of sodium fluoride (NaF), 2 mM of sodium orthovanadate (Na_3_VO_4_), 10 mM of sodium pyrophosphate (NaPPi), 1 mM of phenylmethylsulfonyl fluoride (PMSF), 1 μg/ml of leupeptin, 1 μg/ml of aprotinin, and 100 μg/ml of trypsin inhibitor (Sigma, St. Louis, MO, USA). Fibroblasts’ protein content was determined by the Bradford method, as described above. Fibroblast activation protein (FAP) and α-smooth muscle actin (α-SMA) expression were identified in samples containing 12 and 15 µg of protein (for FAP and α-SMA, respectively) resolved by 7.5% and 12.5% SDS–polyacrylamide gel electrophoresis (SDS-PAGE) (for FAP and α-SMA, respectively) under reducing conditions and with heating. Separated proteins were then blotted onto a nitrocellulose membrane (Whatman-GE Healthcare Life Sciences, London, UK), and non-specific binding sites were blocked for 2 h in 10% non-fat dry milk in TBS containing 0.5% Tween-20 (TBS-T) at room temperature.

Blots were then probed with the specific primary antibody at 4°C overnight: FAP (rabbit monoclonal, 1:1,000 dilution, ab207178, Abcam, Cambridge, UK) and α-SMA (rabbit monoclonal, 1:5,000 dilution, ab32575, Abcam, Cambridge, UK). GAPDH (mouse monoclonal, 1:5,000 dilution; MA5-11114; Thermo Scientific) was used as a normalizer. After several washes in TBS-T, the membranes were incubated in appropriate horseradish peroxidase (HRP)-conjugated secondary Abs: goat anti-mouse IgG-HRP, dilution 1:10,000 (sc-2005, Santa Cruz Biotechnology, Dallas, TX, USA) or goat anti-rabbit IgG-HRP, dilution 1:7,500 (sc-2204, Santa Cruz Biotechnology) for 1 h. All the antibodies were diluted in blocking buffer (TBS-T containing 1% non-fat dry milk). After being washed in TBS-T, the reactive bands were visualized with a chemiluminescence detection kit (SuperSignal West Femto Chemiluminescent Substrate, Thermo Scientific).

Images were recorded and analyzed with the gel documentation system Alliance LD2 (Uvitec, Cambridge, UK).

### Collection of Normal Human Dermal Fibroblast Conditioned Media

To verify if treated NHDF modify their secretome, after the 5 days of cumulative treatment with 1 μg of EVs/ml, cells were washed with serum-free DMEM and then incubated for 24 h in a complete medium in which FBS was replaced with 0.2% Lactalbumin Enzymatic Hydrolysate (LEH; Sigma, St. Louis, MO, USA) to remove the contribution of enzymes/growth factors from the serum. Parallelly, CM was prepared in the same manner from untreated fibroblasts (controls). Cells and cell debris were removed by centrifugation at 600–1,550×*g* from all the CMs. Then, CMs were concentrated using Centricon Ultracel YM-10 filters (Amicon Bioseparations; Millipore Corporations, MA, USA; cutoff, 10 kDa) to be analyzed by casein–plasminogen zymography assays or were used unconcentrated for tests such as proliferation, migration, and invasion assays, in addition to gelatin zymography assays.

Hereinafter, CMs obtained from NHDF, NHDF_30′_, and NHDF_18h_ will be indicated, respectively, as CM NHDF, CM NHDF_30′_, and CM NHDF_18h_.

### Proliferation Assay

NHDF (1,000 cells/well) were seeded onto a 96-well plate, incubated for 24 h in complete medium to enable cell adhesion and spreading, and then treated with EVs_30′_ and EVs_18h_ as explained above (1 µg of EVs/ml every day for 5 days). The effects of EVs on NHDF proliferation were evaluated by the XTT assay on the 5th day, i.e., at 96 h from the beginning of the EV treatment, while treatment was still in progress. Untreated fibroblasts, grown in the same medium but without EVs, were used as control.

For experiments with CM, NHDF (1,000 cells/well), ovarian cancer cells CABA I (1,500 cells/well), and HUVECs (1,000 cells/well) were seeded into 96-well plates (gelatin-coated for HUVECs), allowed to adhere and spread for 24 h at 37°C and 5% CO_2_, and then cultured for 96 h (NHDF) or 72 h (CABA I and HUVECs) with the CM NHDF_30′_ and CM NHDF_18h_. At the end of each specified interval, the proliferation was assessed with the XTT assay.

CMs for experiments on NHDF were supplemented with 1% FBS to ensure fibroblast survival, without stimulating their growth; for the same reason, CMs for HUVEC experiments were supplemented with 5% FBS, 5% NCS, HEPES, heparin, and ECGF; CABA I cells were incubated with unsupplemented CM. Cells incubated with CM NHDF were used as controls.

For the proliferation assay, 1 mg/ml of XTT [2,3-bis(2-methoxy-4-nitro-5-sulfophenyl)-2*H*-tetrazolium-5-carboxamide] (Sigma, St. Louis, MO, USA) and 1.53 mg/ml of phenazine methosulfate (PMS; Sigma-Aldrich) were mixed, and 50 μl of this solution was added to each well. Plates were incubated for 4 h at 37°C, 5% CO_2_; after this interval, the optical density (OD) of the colored, non-toxic, water-soluble formazan originated by the metabolic reduction of XTT mixed with PMS by mitochondria of living cells was measured by an ELISA reader at 450 nm. Values obtained in the absence of cells were considered as background and subtracted from the OD values of the samples. XTT tests were performed before the cells reached confluence to prevent any possible artifact decrease in the results due to contact inhibition.

Each experiment was performed in triplicate and repeated at least twice. The data are expressed as the means ± SDs.

### *In Vitro* Scratch Wound-Healing Assay

The wound-healing assay is one of the earliest developed tests to study directional cell migration *in vitro*, and it is based on the observation of cell migration into a scratch “wound” created on a cell monolayer.

NHDF were cultured in 24-well microplates and treated as previously explained. The scratch was performed at 48 h after the end of 5 days’ treatment with EVs_30′_ and EVs_18h_ when the cells had reached the full confluency; a previously sterilized 200-µl plastic tip was drawn across the cellular stratum to produce a wound, floating cells were removed, and wells were washed 3 times with PBS to remove debris and to smooth the edge of the wound. During the migration into the wound, cells were maintained in an FBS reduced culture medium (2% FBS) that avoided scratch closure by means of cell growth.

The status of the scratch wounds was monitored up to 48 h using a contrast-phase microscope; representative images were collected at the beginning of the assay and at regular intervals. The surface of the wounded area in each image was quantified with the ImageJ software, and the data were reported as % of wound closure (compared to 100%, conventionally assigned to the original scratch area).

### Invasion Assay

The study of cell invasiveness was accomplished using modified Boyden chambers, separating the upper and lower compartments with filters (8-μm pore size polycarbonate polyvinylpyrrolidone-free Nucleopore filters) coated with a thin layer of Matrigel^®^ Growth Factor reduced (Beckton Dickinson, Franklin Lakes, NJ, USA) diluted in serum-free medium to a concentration of 0.5 mg/ml.

Briefly, NHDF, NHDF_30′_, and NHDF_18h_ (1,000 cells/well) were added to the upper chamber in 45 μl of serum-free medium, and their motility abilities were tested using as chemoattractant some DMEM containing 10% FBS, which was added into the lower chamber; NHDF were used as controls.

In experiments with ovarian cancer cells, CABA I cells (1,000 cells/well) were added to the upper chamber in 45 μl of serum-free medium, and in the lower chamber were added the serum-free CM NHDF_30′_ and CM NHDF_18h_ to test their effect as chemoattractant; cells invading in response to CM NHDF were used as controls.

The cells were allowed to invade the Matrigel^®^ for 24 h at 37°C, 5% CO_2_. The non-invading cells on the upper surface of the 8-μm pore filters were removed with a cotton swab. The invading cells on the filters’ lower surface were fixed and stained in 1% crystal violet in methanol. Invading cells in five random microscope fields for each well were counted at 20× magnifications.

### Zymography Assays

Serum-free CM NHDF, CM NHDF_30′_, and CM NHDF_18h_ were subjected to both gelatin and casein–plasminogen zymography assays. Gelatin zymography was performed using 7.5% SDS-PAGE copolymerized with 1 mg/ml of gelatin type B (Sigma, St. Louis, MO, USA); the CMs were diluted in SDS-PAGE sample buffer and analyzed under non-reducing conditions without heating. After electrophoresis, the gels were washed three times, 15 min each, at room temperature, in a washing buffer containing 50 mM of Tris-HCl (pH 7.4) and 2.5% Triton X-100 (Sigma-Aldrich); they were, then, incubated overnight in an activation Tris buffer (50 mM of Tris-HCl, pH 7.4, 5 mM of CaCl_2_, and 120 mM of NaCl) at 37°C. To visualize the lytic bands, the gels were stained with Coomassie Blue R 250 (Bio-Rad, Hercules, CA, USA) dissolved in a mixture of methanol:acetic acid:water (4:1:5) for 30 min and then destained in the same solution without dye.

The plasminogen activators (PAs) in the concentrated culture CM were examined using the casein–plasminogen zymography under non-reducing conditions and without heating. Proteins were separated by electrophoresis in 10% SDS-PAGE copolymerized with 0.2% casein (Sigma-Aldrich, St. Louis, MO, USA) and 10 mg/ml of human plasminogen (Sigma-Aldrich, St. Louis, MO, USA). After electrophoresis, the gel was washed in the same buffer used for the gelatinase assay and then incubated for 48 h at 37°C in 50 mM of Tris-HCl, pH 7.4 + 0.02% NaN_3_. Staining and destaining were performed as previously described. Activities of gelatinases and PAs appeared as clear and distinct bands, which indicated proteolysis of the substrate, on a blue background: those digestion bands were quantified by ImageJ software.

### Electron Microscopy

Scanning electron microscopy (SEM) analysis was performed on fibroblasts treated with EVs_30′_ and EVs_18h_ for up to 5 days. Forty-eight hours after the end of this treatment, NHDF, NHDF_30′_, and NHDF_18h_ were detached, washed, and allowed to grow to subconfluence on coverslips for an additional 96 h; then cells were fixed in 2% glutaraldehyde (Electron Microscopy Sciences, Hatfield, PA, USA) in PBS for 3 min.

After being dehydrated with a graded scale of ethanol (30% to 100%) and critical point-dried, the samples were glued onto stubs, coated with gold in an SCD040 Balzer Sputterer, and detected with Philips 505 SEM at 20 kV.

### Migration Assay

The migration of normal fibroblasts and CABA I cells was tested in response to CM NHDF_30′_ and CM NHDF_18h_ (added as a chemoattractant in the lower chambers, the same volume for each sample). Cells migrated in response to CM NHDF were used as controls. Briefly, cells were detached, washed three times in serum-free medium, and seeded on the upper wells (5,000 cells/wells in serum-free medium) of the modified Boyden chamber. Gelatin-coated polycarbonate membranes with 8-µm pores were used to separate the upper wells from the lower ones. Each condition to be tested was analyzed in triplicate. The Boyden chambers were incubated for 24 h at 37°C in a CO_2_ incubator, and then migrated cells were visualized as described for the invasion assay. The number of cells, migrated to the lower surface of the polycarbonate membrane, was counted in five random 20× fields within each well, under a microscope. The mean number of cells per field was calculated as cell counts.

### Tube Formation Assay

This *in vitro* test measures the ability of endothelial cells to invade, migrate, organize, and differentiate into capillary-like tubular structures within a three-dimensional matrix constituted by Matrigel^®^ Growth Factor Reduced 10 mg/ml (BD56230, Franklin Lakes, NJ, USA). Briefly, Matrigel^®^ was plated on the bottom of 96-well plates and allowed to gel at 37°C for 1 h. HUVECs were detached, counted, washed in serum-free medium, and resuspended in serum-free CM NHDF, CM NHDF_30′_, and CM NHDF_18h_. Then, 20,000 cells/well were seeded on Matrigel^®^ and incubated at 37°C, 5% CO_2_; the tube formation was observed at 7 h after cell seeding. Several images were acquired per well and processed using the Angiogenesis Analyzer plugin with ImageJ software (28) downloadable from the National Institutes of Health website. The total length of the capillary-like structures, the number of nodes, and the number of segments normalized per area were used for data analysis.

### Statistical Analysis

Data are expressed as the mean ± standard error. Comparisons between the means of control groups and treated groups were performed using the one-way ANOVA followed by Tukey’s post-test; results were considered statistically significant when p < 0.05 (*), p < 0.01 (**).

## Results

### Normal Fibroblasts Treated With Extracellular Vesicles Acquire Cancer-Associated Fibroblast-Like Morphologyand Express Their Markers

Potential NHDF morphological changes, a typical signature of fibroblast activation, induced by EVs_30′_ and EVs_18h_ were observed by an inverted optical microscope.

EVs_30′_ and EVs_18h_ are EVs isolated from CMs collected after 30 min and 18 h, respectively. As mentioned above and discussed further below, in previous work (25), we highlighted that the human ovarian cancer cell line CABA I releases two specific subpopulations of sEVs “(EVs30’) and lEVs+sEVs (EVs18h).

**Figure 1:**
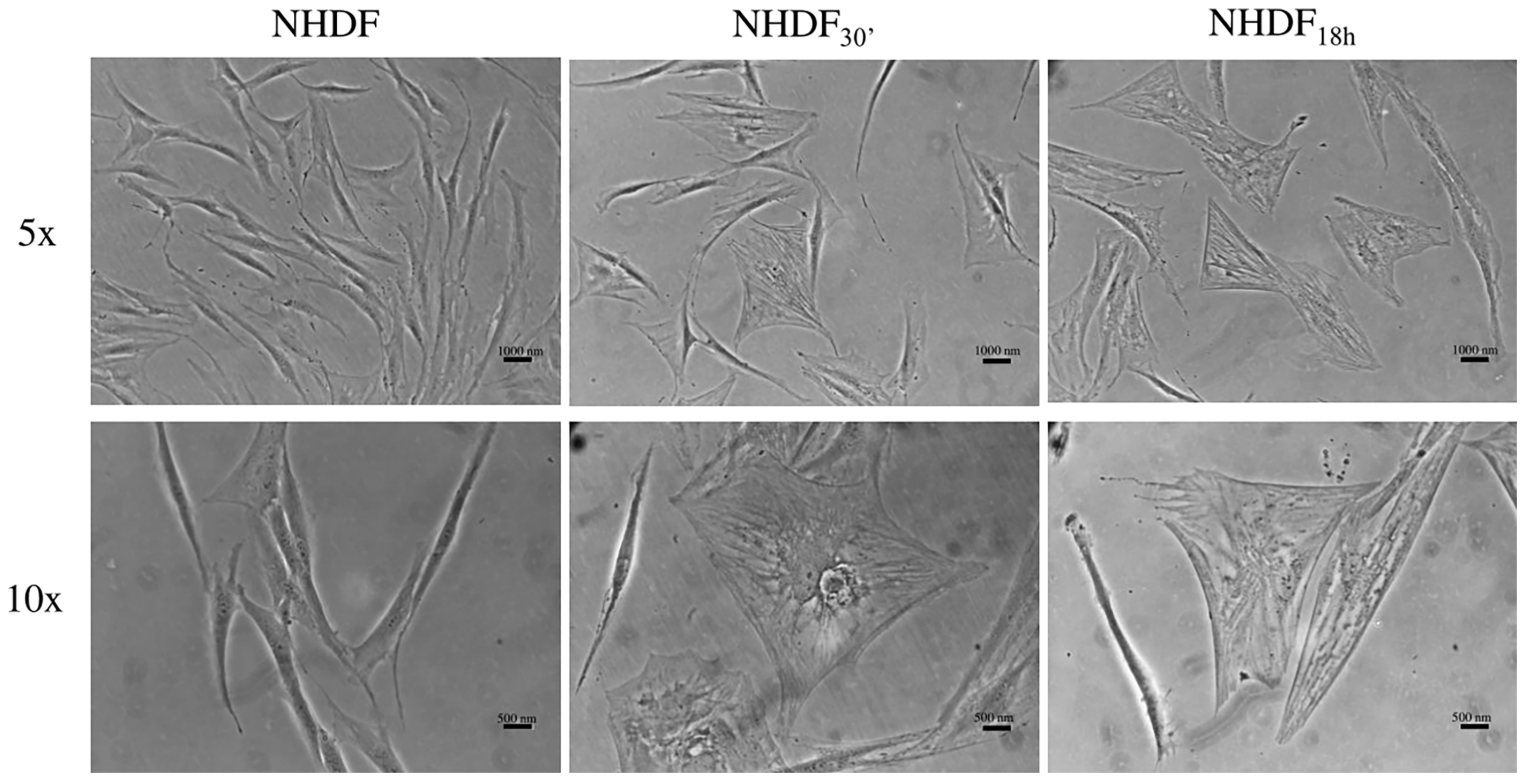
Optical images of fibroblasts showing morphological changes induced by extracellular vesicle (EV) subpopulations EV_30′_ and EV_18h_. Representative images of untreated control fibroblasts (NHDF) and fibroblasts treated with the two EV-subpopulations (NHDF_30′_ and NHDF_18h_). The scale bar is 1,000 nm in the top row and 500 nm in the bottom row. Images were captured with 5× and 10× objectives of an inverted optical microscope.

Such morphological changes were visible in NHDF treated with ovarian cancer EVs_30′_ and EVs_18h_ starting after 72 h at the beginning of treatment (**Figure 1**), whilst untreated fibroblasts exhibit typical elongated and spindle-shaped morphology, some NHDF_30′_ and NHDF_18h_ underwent a morphological change, acquiring the typical morphology of activated fibroblasts (NHDF_30′_ and NHDF_18h_ are, respectively, NHDF treated with EVs_30′_ and EVs_18h_): they appeared very spread with many visible stress-contractile fibers inside the cytoplasm.

**Figure 2:**
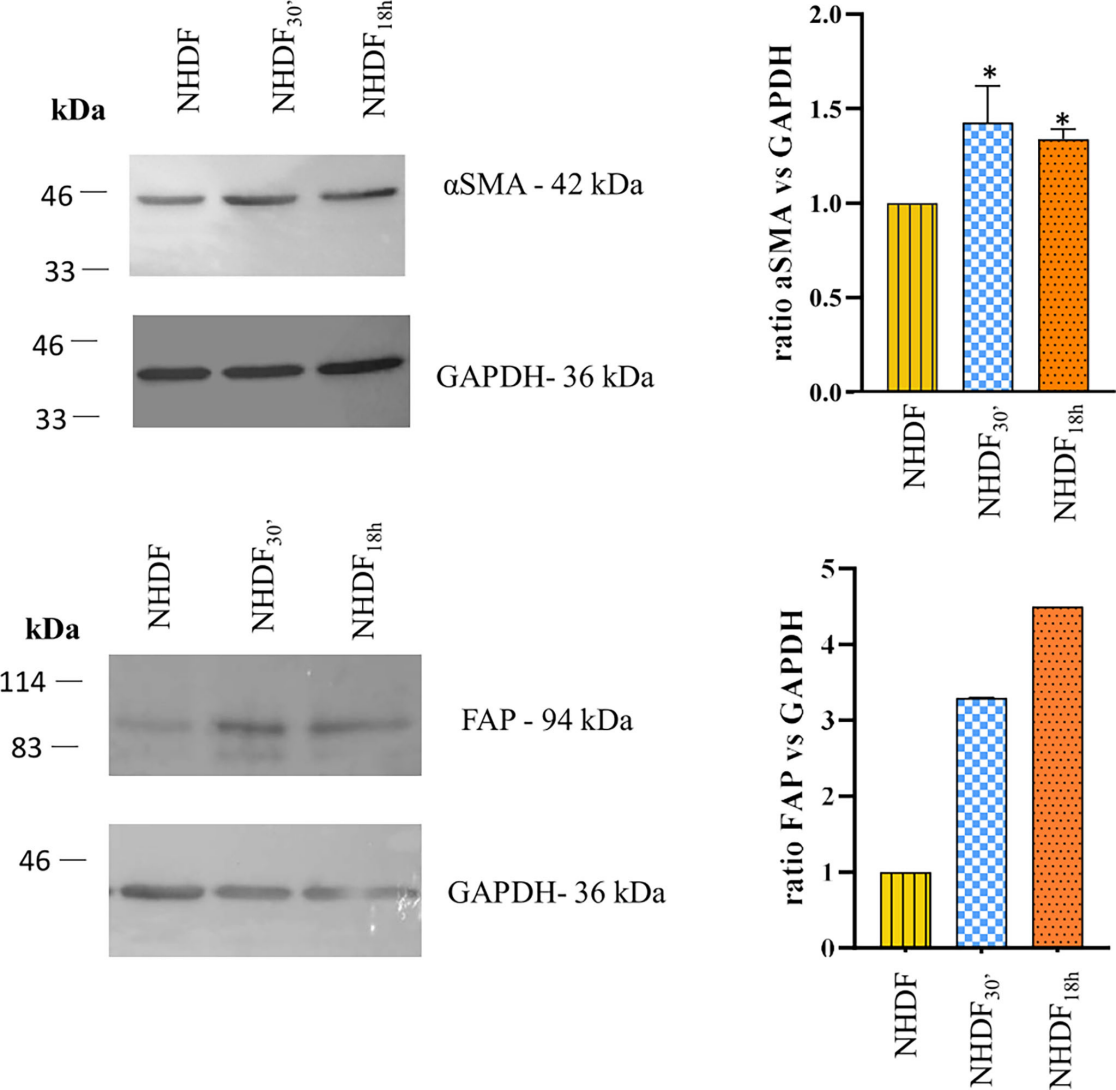
Western blotting identification of α-smooth muscle actin (α-SMA) and fibroblast activation protein (FAP). The expression of α-SMA and FAP was increased in NHDF_30′_ and NHDF_18h_. Band intensity was analyzed by ImageJ and presented in the graph on the right as ratio α-SMA/GAPDH or FAP/GAPDH, in which 1 is the ratio conventionally attributed to NHDF. For α-SMA, the image on the left is representative of 1 of 3 independent experiments (all of them represented in the graph as mean ± SD; *p < 0.05). FAP assay was performed once.

To confirm the cell activation, at the end of the EV treatment, NHDF, NHDF_30′_, and NHDF_18h_ were lysed as described, and protein extracts were analyzed to detect the expression of typical markers of CAFs: FAP and α-SMA (**Figure 2**).

The quantitative analysis detected a statistically significant increase in the expression of α-SMA (calculated molecular weight: ~44 kDa) in both NHDF_30′_ and NHDF_18h_ when compared to NHDF (1.44 and 1.35, respectively) (**Figure 2**). FAP was also increased in both NHDF_30′_ and NHDF_18h_ when compared to NHDF (3.3 and 4.5, respectively).

### Extracellular Vesicle Subpopulations Differently Affect Fibroblast Proliferation, Motility, Invasiveness, Enzyme Expression, and Microvesicle Release

**Figure 3:**
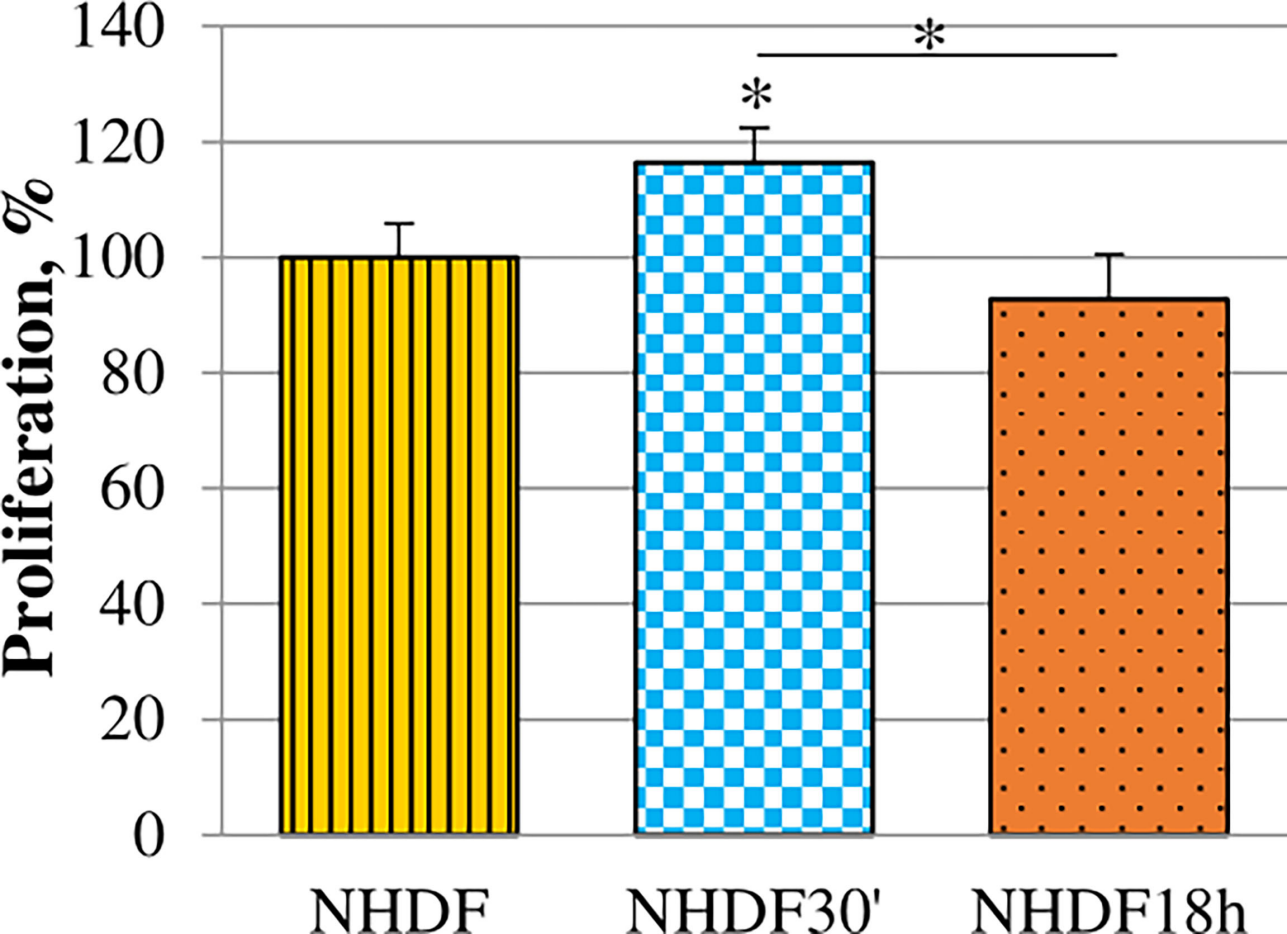
Effects of EVs_30′_ and EVs_18h_ on fibroblasts’ proliferation. Proliferation was evaluated by XTT assay, on the 5th day of treatment, i.e., at 96 h from the beginning of the EV treatment. Values were calculated as mean ± SD and are expressed as percentages with respect to 100% proliferation, conventionally attributed to untreated NHDF. Experiments were performed three times in triplicate. The asterisk on the bar indicates the statistical significance with respect to NHDF, and the horizontal line refers to the statistical significance between the NHDF_30′_ and NHDF_18h_ (*p < 0.05).

Proliferation rate alteration induced by EV treatments was evaluated. It was tested while the treatment was still ongoing on the 5th day of the EV treatment (96 h from the beginning of treatments) (**Figure 3**): EVs_18h_ did not induce any significative increase, while the treatment with EVs_30′_ resulted in a significant increase when compared to the untreated cells NHDF (+15%).

**Figure 4:**
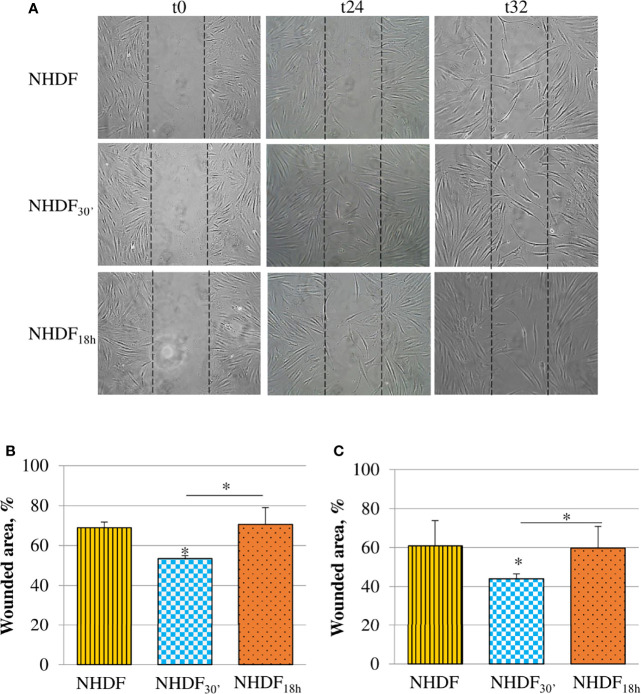
Effects of EVs_30′_ and EVs_18h_ on fibroblasts’ motility. Motility was assessed by the scratch wound assay **(A)**; the panel reports representative images recorded 24 and 32 h after the scratch creation (0 h); dotted lines represent the size of the original wound. Graphs at the bottom show the percentage of the still wounded area at 24 h **(B)** and 32 h **(C)** with respect to the original wound (conventionally set as 100%). Only the migration of fibroblasts treated with EVs_30′_ resulted in statistical significance compared to the migration of control fibroblasts. Data derived from three biological replicates tested individually due to scarcity of the material and are shown as mean ± SD; the asterisk on the bar indicates the statistical significance with respect to NHDF, and the horizontal line refers to the statistical significance between the NHDF_30′_ and NHDF_18h_ (*p < 0.05).

The motility induced by EVs_30′_ and EVs_18h_ treatments was tested with the scratch wound assay (**Figure 4**). Migration was observed at different time intervals (24, 32, and 48 h), and the most significant changes were captured after the beginning point (time zero); the observation of the wounded area showed that NHDF_30′_ and NHDF_18h_ have a greater tendency to close the wound by migrating inside it compared to NHDF (**Figure 4A**); to quantify this ability to close the wound, the wounded area (i.e., the area uncovered from the cells) was measured with ImageJ software at the intermediate time intervals, 24 h (**Figure 4B**) and 32 h (**Figure 4C**). The 100% value was conventionally assigned to the wounded area of the original scratch (time zero). NHDF_18h_ migrated with the same trend as NHDF, while NHDF_30′_ exhibited higher motility: indeed, after 24 h, the area not yet covered was quite comparable in NHDF and NHDF_18h_ (respectively 69% and 71% with respect to the original wound), but it was significantly lower (53% with respect to the original wound) in NHDF_30′_. After 32 h, the scratch area of NHDF and NHDF_18h_ was again comparable (respectively 61% and 60% with respect to the original wound), but it was significantly lower in NHDF_30′_ (44% compared to the original wound). After 48 h, the trend was substantially maintained, but since proliferative effects could begin to occur at this time interval, it was not considered (despite that the experiment conducted in the presence of a low concentration of serum has certainly avoided the scratch closure by means of cell growth) (data not shown).

**Figure 5:**
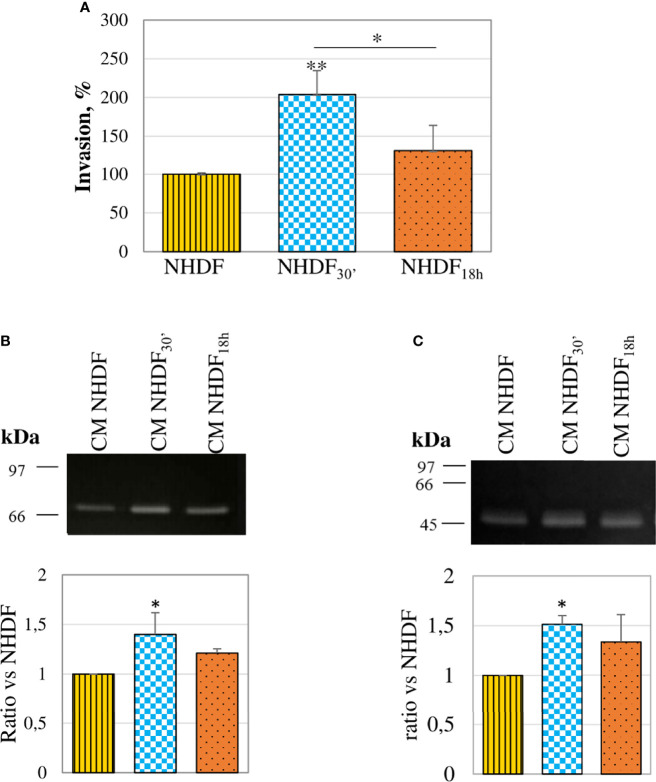
Invasion ability and proteolytic enzymes. **(A)** In an invasion assay with a modified Boyden chamber, NHDF_30′_ and NHDF_18h_ invaded through the Matrigel^®^-coated membrane significantly more than NHDF. Data derive from 5 measures from each replicate (3 replicates in total) and are shown as mean ± SD; the asterisk on the bar indicates the statistical significance with respect to NHDF, and the horizontal line refers to the statistical significance between the NHDF_30′_ and NHDF_18h_ (*p < 0.05; **p < 0.01). **(B)** Gelatin zymography assay was performed to detect gelatinolytic activity in the serum-free conditioned media of NHDF, NHDF_30′_, and NHDF_18h_. EVs_30′_ induced a more marked increase in pro-MMP-2 (~72 kDa) release than the EVs_18h_. **(C)** Casein–plasminogen zymography assay was performed to detect plasminogen activator (PA) activity in the serum-free conditioned media of NHDF, NHDF_30′_, and NHDF_18h_. The bands represent the high-molecular-weight PAs (~48-55 kDa), whose release resulted in higher fibroblasts treated with EVs_30′_. In both zymography assays, the densitometric values of the bands were calculated with ImageJ and reported in the graphs below as a ratio of the band NHDF_30′_ or NHDF_18h_ vs. NHDF, which have been conventionally assigned the value 1. The images shown in panels B and C are representative of 3 independent experiments (all of them being reported in the graphs).

The invasion assay performed with the modified Boyden chamber showed that both fibroblasts treated with EVs_30′_ and EVs_18h_ showed a trend to a greater invasiveness capacity (respectively +101% and +30%) as compared to NHDF (**Figure 5A**), but only NHDF_30′_ had a statistically significant greater ability if compared to NHDF. To estimate if the invasion ability induced by the EV treatment could be supported by an increased secretion of proteolytic enzymes, CMs from EV-treated fibroblasts were normalized according to the same volume and assayed to evaluate the gelatinolytic and PA activities by employing zymographic techniques. The gelatinase assay (**Figure 5B**) revealed that both EVs_30′_ and EVs_18h_ induced in NHDF the expression of pro-MMP-2: +41% and +24%, respectively, in NHDF_30′_ and NHDF_18h_ compared to NHDF (calculated molecular weight 70 kDa). The casein–plasminogen zymography (**Figure 5C**) similarly highlighted a trend to a higher release of the high-molecular-weight urokinase-type PA (HMW-uPA) (calculated molecular weight 48–55 kDa), particularly in NHDF_30′_ (+51% in NHDF_30′_ compared to NHDF).

**Figure 6:**
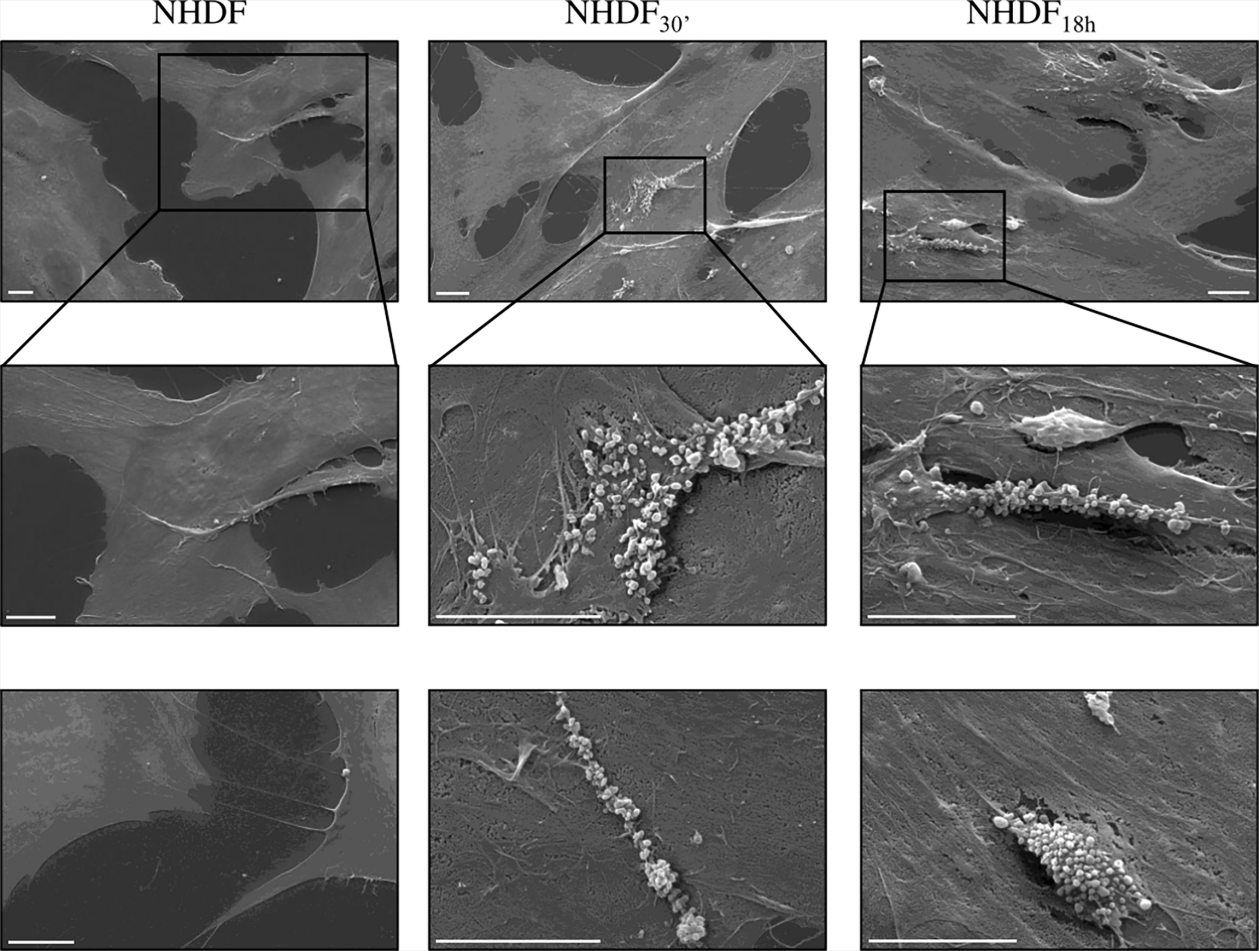
Scanning electron microscopy (SEM). SEM images highlighted the intense shedding of microvesicles from the plasma membrane of NHDF_30′_ and NHDF_18h_. On the other hand, NHDF cells showed an extremely sporadic production of microvesicles. The first row shows images at low magnification; the second row shows details of the first row at higher magnification, as highlighted by the boxes; row 3 shows other independent and representative images at higher magnification. The scale bar is 10 µm in all images.

NHDF_30′_ and NHDF_18h_ cell surface was also observed by SEM to verify if EV-mediated activation stimulated, in turn, the EV release, particularly of MVs from the cell surface, whilst the shedding of MVs was very sporadic in untreated NHDF; in EV-treated fibroblasts, the extent of the MV release was more evident and involved large membrane areas (**Figure 6**).

### Secretome of NHDF_30′_ and NHDF_18h_ Affects Bystander Cells

After the end of the EV treatment, the CMs of NHDF_30′_ and NHDF_18h_ (representing the cell secretome and containing both EV-associated and soluble molecules) were used as stimuli to evaluate their effect on the cells normally present in the tumor microenvironment, such as fibroblasts, endothelial cells, and tumor cells. CM from untreated NHDF was used as a control.

**Figure 7:**
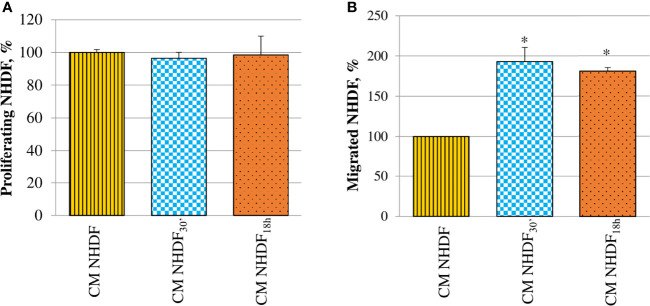
Effect of NHDF, NHDF_30′_, and NHDF_18h_ secretome on normal fibroblasts. **(A)** Normal fibroblasts were cultured for 96 h with conditioned media from NHDF, NHDF_30′_, and NHDF_18h_. Cell proliferation rate was tested using XTT assay. Data were derived from experiments performed twice in triplicates and are shown as mean ± SD. No significant differences in the proliferation percentage were revealed. **(B)** Normal fibroblast migration assay performed twice in duplicates with modified Boyden chamber. Data are expressed as mean ± SD and are shown as a percentage with respect to 100% migration, conventionally attributed to fibroblasts migrated in response to conditioned medium of NHDF (*p < 0.05).

Fibroblast proliferation rate was not at all affected by CM (**Figure 7A**). On the contrary, CM NHDF_30′_ and CM NHDF_18h_ exerted a considerable chemotactic effect, stimulating the migration ability of normal fibroblasts (**Figure 7B**): migration of fibroblasts toward the secretome of EV-treated fibroblasts was almost 2-fold increased with respect to the migration toward the CM NHDF (+93% and + 81%, respectively for CM NHDF_30′_ and CM NHDF_18h_), even if no differences were appreciable between CM NHDF_30′_ and CM NHDF_18h_.

**Figure 8:**
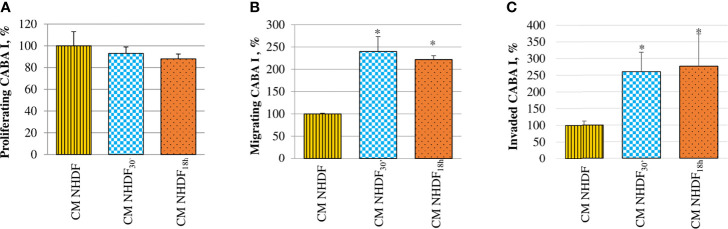
Effect of NHDF, NHDF_30′_, and NHDF_18h_ secretome on CABA I cells. **(A)** CABA I cells were cultured for 72 h with conditioned media (CMs) of NHDF, NHDF_30′_, and NHDF_18h_. Data, derived from experiments performed twice in triplicates, are expressed as mean ± SD and shown as a percentage, and 100% proliferation was assigned to CABA I cells proliferating with CM of untreated NHDF. **(B)** CABA I cell migration was measured twice in duplicate in response to serum-free CM NHDF, CM NHDF_30′_, and CM NHDF_18h_, by modified Boyden chamber (*p < 0.05). Data (mean ± SD) are expressed as a percentage with respect to 100% migration, conventionally attributed to CABA I cells migrating toward CM NHDF. **(C)** CABA I cell invasion was tested twice in response to CM NHDF, CM NHDF_30′_, and CM NHDF_18h_ with a modified Boyden chamber coated with Matrigel^®^ (*p < 0.05). Data (mean ± SD) are expressed as a percentage, and 100% invasion was attributed to CABA I cells migrating toward CM NHDF.

The effects of activated fibroblasts’ secretome on ovarian cancer cells were also analyzed evaluating the migration and invasion abilities, in addition to their proliferative capacity (**Figure 8**). CABA I cells cultured in the presence of CM NHDF, CM NHDF_30′_, and CM NHDF_18h_ showed no significant change in their proliferation (**Figure 8A**). On the contrary, their motility (**Figure 8B**) and invasiveness (**Figure 8C**) were significantly promoted: they were higher in response to CM NHDF_30′_ and CM NHDF_18h_ than in response to CM NHDF, with no significant differences between CM NHDF_30′_ and CM NHDF_18h_ (motility, +140% and +116% compared to CM NHDF in CM NHDF_30′_ and CM NHDF_18h_, respectively; invasion, +158% and +176% compared to CM NHDF in CM NHDF_30′_ and CM NHDF_18h_, respectively).

**Figure 9:**
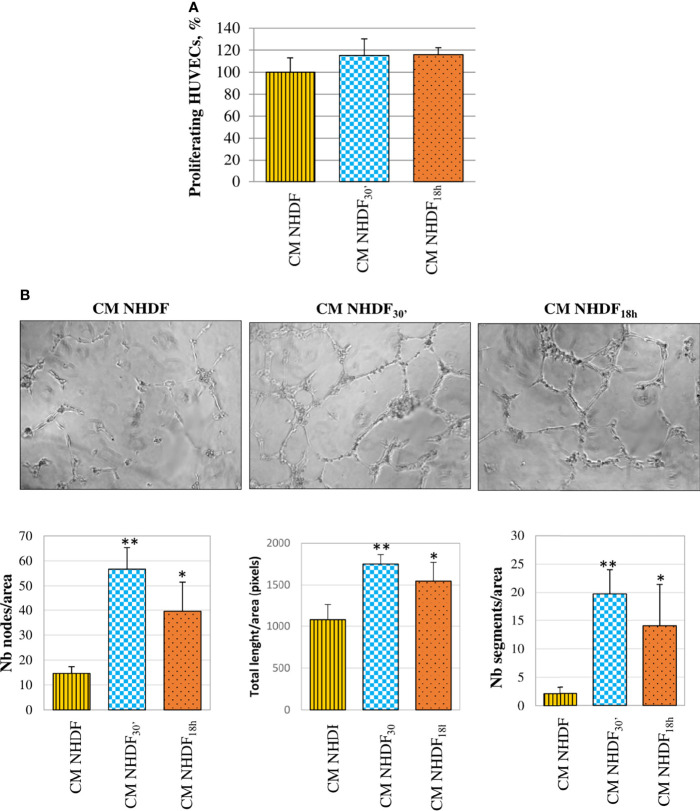
Effect of NHDF, NHDF_30′_, and NHDF_18h_ secretome on HUVECs. **(A)** The effect of conditioned media (CM) NHDF, CM NHDF_30′_, and CM NHDF_18h_ on endothelial cell growth was assessed using the XTT assay. Proliferation was expressed as a percentage, conventionally attributing 100% proliferation to HUVECs treated with CM NHDF. Data derived from experiments performed twice in triplicates. **(B)** Representative pictures showing the formation of capillary-like structures formed by HUVECs seeded on Matrigel^®^-coated plates in a serum-free condition and treated with CM NHDF, CM NHDF_30′_, and CM NHDF_18h_; the graphs at the bottom show the number of nodes or total length of tubes or number of segments normalized per area. Data derive from experiments performed twice in duplicate and are expressed as the mean ± SD (*p < 0.05; **p < 0.01).

HUVECs, too, were stimulated by CM NHDF, CM NHDF_30′_, and CM NHDF_18h_ to assess their proliferation response (**Figure 9**): although the cell number appeared to increase in endothelial cells treated with CM from EV-treated fibroblasts, this increase was not statistically significant (**Figure 9A**). On the contrary, the tube formation assay highlighted the pro-angiogenic potential of CM. The test revealed that the differentiation of HUVECs into primitive capillary-like structures occurred in response to both CM NHDF_30′_ and CM NHDF1_8h_; the number of nodes, the total length of formed tubes, and the number of segments were significantly higher in HUVECs treated with CM NHDF_30′_ and CM NHDF_18h_ than with CM NHDF (**Figure 9B**) (number nodes/area: 14.6 in HUVECs treated with control NHDF CM; 56.7 and 39.6 in CM NHDF_30′_- and CM NHDF_18h_-treated HUVECs, respectively. Total length/area: 1,078 pixels in HUVECs treated with control CM NHDF; 1,751.6 and 1545.8 pixels in CM NHDF_30′_ and CM NHDF_18h_ treated HUVECs, respectively. Number segments/area: 2.12 in HUVECs treated with control CM NHDF; 19.7 and 14 in CM NHDF_30′_ and CM NHDF_18h_ treated HUVECs, respectively).

## Discussion

Ovarian cancer is one of the deadliest gynecological malignancies and is characterized by a poor prognosis, with an overall 5-year survival rate lower than 40%, which increases when the cancer is diagnosed at an early stage—while still confined to the ovary—and treated by surgery and chemotherapy (29, 30). Many cases of ovarian cancer, unfortunately, are diagnosed when already in an advanced stage, with metastasis to bladder, uterus, or abdomen, as ovarian cancer symptoms typically resemble gastrointestinal problems (abdominal discomfort, nausea, and bloating) (20). The traditional clinical approach to ovarian cancer relies on a combination of surgery and platinum/taxane-based chemotherapies. While initially sensitive to chemotherapeutic drugs, unfortunately, most patients develop a resistance to these pharmacological therapies (30).

So far, the used therapeutic drugs predominantly targeted the tumor cells, without taking proper account of the role of the tumor microenvironment. The latter, instead, is composed of an extracellular matrix and many cells that could actively participate in tumor progression and may serve as novel therapeutic targets for ovarian cancer patients (20, 21, 31). Among the stromal cells—besides adipocytes, endothelial cells, and immune cells—fibroblasts have been strongly reconsidered, as their ability to create a loop of intercellular communications that strengthen the cancer progression has been revealed (31).

It has been highlighted, indeed, that within the tumor microenvironment, fibroblasts, which usually constitute the most abundant population, can acquire a perpetually “activated” state, making them able to support, in turn, the cancer progression; these activated fibroblasts were identified as CAFs (20, 32–34). Besides, CAFs’ supportive role in ovarian cancer has been already proved (21, 35, 36).

Generally, the activation of resident fibroblasts, induced by the cross-talk with tumor cells, may be sustained by growth factors released from tumor cells, the most important being the TGF-β, even if many other molecules seem to be involved in the CAFs activation, such as HGF, PDGFs, FGFs, EGFs, and interleukin-1β (18, 30, 37, 38). Once activated from cancer cells, CAFs’ secretome, in turn, remodels tumor stroma to become more advantageous for tumor progression, thus deeply contributing to the malignant behavior of cancer cells. CAFs, indeed, can enhance the invasive properties of cancer cells releasing several tumor-promoting growth factors and chemokines (for example, TGF-β, HGF, FGF1, and FGF2) and also molecules (like VEGF) that strongly induce angiogenesis, further supporting proliferative, migratory, and invasive abilities of cancer cells (29, 33, 35, 39–41). They can migrate along with cancer cells in the bloodstream, secreting cytokines that sustain invasive properties and growth of tumor cells at distant sites, supporting the hypothesis that they can contribute to the pre-metastatic niche formation; they also support immunosuppression and drug resistance (22–24, 37, 38, 41–43).

As for the latter, the key role of CAFs and how they exploit several mechanisms to sustain the resistance to antineoplastic drugs have emerged: they can modify the composition of the extracellular matrix so to increase the intratumoral interstitial fluid pressure, resulting in a physical barrier that prevents an efficient delivery of anticancer drugs (22, 44). CAFs can also activate signaling pathways that revert the therapeutic outcome, driving tumor cells to a more chemoresistant phenotype by different mechanisms (45): they can release growth factors involved in the therapy resistance (among the growth factors released from CAFs, for example, the HGF has been correlated to therapeutic resistance occurrence in melanoma) (22, 46) or factors that, stimulating tumor cells to undergo epithelial-to-mesenchymal transition, increase the resistance to chemotherapy (24, 45); they have also been shown to promote the chemoresistance by promoting the metabolic reprogramming or maintaining the stemness of cancer stem cells (23).

Being increasingly demonstrated that CAFs contribute to cancer progression and drug resistance, they are more and more considered as a pivotal target of novel anticancer therapies. In parallel, the understanding of biological processes involved in CAFs activation into the tumor microenvironment is critical to reveal mechanisms underlying cancer progression and drug resistance as well.

Since EVs are known for their role as mediators of cell-to-cell communication (3, 47–49), we wondered if EVs released from human ovarian cancer cells could activate normal fibroblasts into CAFs, as is the case with other types of cancer (18, 50–54) and found out that when EVs isolated from human ovarian CABA I cancer cells were administered to normal fibroblasts, they induced their activation into a CAF-like state (55).

Moreover, we had already demonstrated that it is possible to isolate two EV subpopulations from CABA I cells in a time-dependent way: starved CABA I cells, once stimulated with FBS, released a nearly pure population of EXO-like EVs or sEVs (mean size ~100 nm) after 30 min and a second population consisting of a high amount of MVs-like EVs or lEVs (size > 100 nm) combined with a low EXO-like EV contribution, i.e., lEVs+sEVs after 18 h (25). Those data highlighted that different time intervals lead to the release of different subpopulations of EVs, in terms of not only size but also amount and molecular composition, suggesting possible different cargoes and, consequently, a different biological role for the different subpopulations.

Hereinafter, these subpopulations will be indicated, respectively, as EVs_30′_ and EVs_18h_ and the NHDF cells obtained by their administration as NHDF_30′_ and NHDF_18h_; untreated fibroblasts will be indicated as NHDF.

Based on those previous results, in the present work, we aimed to verify if the two specific subpopulations of sEVs and lEVs+sEVs released from the human ovarian cancer cell line CABA I could differentially activate fibroblasts, so as to verify if they could induce different biological processes (maybe related to a different cargo). To this purpose, NHDF were treated daily with the EV subpopulations for 5 days, in a cumulative way (i.e., adding the new dose of EVs to the previous one without replacing medium throughout the treatment), to reproduce, *in vitro*, continuous stimulation from cancer cells–EVs on stromal fibroblast that, supposedly, takes place *in vivo*.

When administered to fibroblasts, the EVs modified their morphological and molecular features, supporting the idea that EVs can induce the activation of fibroblasts into a CAF-like state: in fact, untreated cells displayed the usual elongated and spindle-shaped aspect of normal quiescent fibroblasts, while some NHDF_30′_ and NHDF_18h_ acquired the typical “spread” phenotype of CAFs, which is similar to that of myofibroblasts involved in the wound-healing process (**Figure 1**) (33, 41); at the same time, there was an increase in the α-SMA levels (**Figure 2**), a common marker for CAFs [along with SDF-1, FSP-1, vimentin, desmin, tenascin, and FAP (20, 33, 41, 56)]. To further confirm the activation into a CAF-like state, FAP was also analyzed, highlighting an increase in its level in EV-treated NHDF.

Even if both EV subpopulations affected the morphology and marker expression in NHDF, we found out that EVs_30′_, but not EVs_18h_, also enhanced the proliferative, migratory, and invasive abilities of NHDF (**Figures 3**–**5**); all these processes are typically increased in CAFs (30, 54). Since NHDF_30′_ releases a higher content of proteolytic enzymes as compared to NHDF_18h_ and NHDF, we can suppose that both invasion and motility were sustained by the increased levels of gelatinases and PAs (**Figures 5B, C**). The release of proteolytic enzymes could also sustain the drug resistance: CAFs have been demonstrated to be actively involved in the secretion of uPA, which can cleave and activate several MMPs that, in turn, could facilitate cancer cells migration and invasion, by degrading the extracellular matrix, as well as drug resistance (18, 24, 33, 34, 57–59). So our data confirm that EV-activated fibroblasts release both the MMPs required for these processes, in an active pro-MMP form, and their activators PAs.

The activated state of NHDF_30′_ and NHDF_18h_ also seems to result in an increased release of EVs (specifically MVs) from the cell surface (**Figure 6**); it is not possible to quantify the extent of MVs’ release from activated NHDF through the SEM images, but the observation clearly revealed an increase in membrane shedding. It has been previously reported that the extensive production of MVs by CAFs is used as a way to move lipids and proteins to target cancer cells to support tumor growth (60). This evidence led to hypothesize that activated fibroblasts are more prone to communicate with neighboring cells; after all, several studies already suggested that CAFs can actively modulate bystander cells in the tumor microenvironment by means of soluble or EV-associated mediators [fibroblasts-derived EXOs, for example, stimulate motility in breast cancer cells (61), while CAF-derived EXOs can lead to higher drug resistance (62)].

This considered, we wondered whether our EVs_30′_- and EVs_18h_-activated fibroblasts were actually able to modulate the response of some cells usually present in the tumor microenvironment, such as tumor and endothelial cells as well as still quiescent fibroblasts. For purely technical problems, due to material shortage, we have not used the EVs isolated from activated fibroblasts but their CM (which represents their whole secretome, containing both soluble and EV-associated molecules). CMs obtained from NHDF, NHDF_30′_, and NHDF_18h_ are indicated, respectively, as CM NHDF, CM NHDF_30′_, and CM NHDF_18h_.

CM NHDF_30′_ and CM NHDF_18h_ did not significantly affect the proliferation, neither of normal fibroblasts (**Figure 7A**) nor tumor (**Figure 8A**) or endothelial cells (**Figure 9A**); on the other hand, instead, both CMs significantly affected the motility of fibroblasts (**Figure 7B**) and motility and invasiveness of CABA I tumor cells (**Figures 8B, C**). The CM from activated fibroblasts also exhibited a pro-angiogenic behavior, being able to stimulate the tube formation assay of HUVECs (**Figure 9B**).

These assays indicated that the secretome released by fibroblasts, being previously activated by cancer EVs, may deeply affect the behavior of neighboring cells through paracrine mechanisms; this observation parallels what is already known for tumor-derived secretome/EVs. Indeed, the role of cancer cells in inducing the reprogramming of other neighboring cells (such as epithelial cells or mesenchymal stem cells) toward a tumor-like phenotype possibly sustaining cancer progression has been already shown (63–69).

Likewise, it is widely demonstrated that tumor EVs can move in the blood, thus contributing to the formation of the pre-metastatic niche (19, 68–70); among the processes involved in the formation of the pre-metastatic niche, a critical role is sustained by the cross-talk between cancer cells and resident fibroblasts, resulting in the activation of the latter ones (18, 71). While many studies dissected the role of tumor-derived EVs in metastatic niche modulation (70–72), only sporadic studies have explored the ability of CAF-derived EVs to promote the pre-metastatic niche formation (73), and their role remains to be further elucidated. Given that the EVs_30′_- and EVs_18h_-activated fibroblasts showed an increased ability to produce MVs and to stimulate, in turn, other normal fibroblasts, our data could support the hypothesis, to be verified, that also the EVs released by the activated fibroblasts in the primary site of the tumor, as well as those released by the tumor cells themselves, can move through the blood and prepare the pre-metastatic niche by stimulating the resident cells.

The disclosed data, overall, support the idea that ovarian cancer cells could initially modulate fibroblast behavior within the tumor microenvironment through the release of EVs, activating them to a CAF-like state, and then, in turn, these CAF-like cells can stimulate the surrounding normal and tumor cells to acquire a cancer-supportive behavior and, maybe, distant fibroblasts in the pre-metastatic niche.

It is interesting to note that the population EVs_30′_ is the strongest in the activation of all described processes, aligning with some proteogenomic assays that have previously shown that EXOs and MVs are functionally distinct (74), with EXOs being more oncogenic than MVs (75); it looks like in the EVs_18h_ population, being the EXOs diluted by the simultaneous presence of MVs, oncogenic stimuli are weakened. The higher ability of EVs_30′_ to activate fibroblast could rely on their higher content in TGF-β with respect to the EVs_18h_, as demonstrated by Western blotting and ELISA (data not shown); TGF-β, along with other several molecules, is required for the induction and maintenance of CAFs by cancer cells (54, 76, 77).

That the EXOs play a crucial role in cancer biology and metastasis has widened their possible applications for cancer detection and medical diagnostics; indeed, there is a continuous evolution of techniques and applications in these fields based on EXO use, ranging from liquid biopsy to EXO-based biosensors (78–80).

There is no doubt that to understand more fully the molecular protagonists of this virtuous (from the tumor point of view) cross-talk, it will be necessary to dissect the content of the EVs_30′_ and EVs_18h_ and the composition of the CAFs secretome; regarding the latter, understanding whether the molecules involved in the stimulation of neighboring cells are soluble or EV-associated could help in identifying involved pathways as well as possible specific therapeutic targets to improve clinical approaches aimed to slow down cancer progression and overcome CAF-supported drug resistance.

## Data Availability Statement

The raw data supporting the conclusions of this article will be made available by the authors, without undue reservation.

## Author Contributions

IG and MDF contributed to conception and design of the study. IG wrote the first draft of the manuscript. IG, MDF, GP, LE, SDA, and VD acquired and analyzed data. IG and VD supervised. VD acquired funds. All authors listed contributed to manuscript revision, read, and approved the submitted version.

## Funding

This study was partly funded by the Department of Life, Health and Environmental Sciences, University of L’Aquila, Italy (RIA 2015-2016).

## Conflict of Interest

The authors declare that the research was conducted in the absence of any commercial or financial relationships that could be construed as a potential conflict of interest.

## Publisher’s Note

All claims expressed in this article are solely those of the authors and do not necessarily represent those of their affiliated organizations, or those of the publisher, the editors and the reviewers. Any product that may be evaluated in this article, or claim that may be made by its manufacturer, is not guaranteed or endorsed by the publisher.

## Abbreviations

α-SMA, α-smooth muscle actin; ABs, apoptotic bodies; CAFs, cancer-associated fibroblasts; CM, conditioned medium; ECGF, endothelial cell growth factor; EGFs, epidermal growth factors; EVs, extracellular vesicles; EXOs, exosomes; FAP, fibroblast activation protein; FBS, fetal bovine serum; FGFs, fibroblast growth factors; HGF, hepatocyte growth factor; HUVECs, human umbilical vein endothelial cells; lEVs, large extracellular vesicles; MMPs, matrix metalloproteinases; MVs, microvesicles; NCS, newborn calf serum; NHDF, normal human dermal fibroblasts; PDGFs, platelet-derived growth factors; SEM, scanning electron microscopy; sEVs, small extracellular vesicles; TGF-β, transforming growth factor β; VEGF, vascular endothelial growth factor.
